# Short-period scattering-assisted terahertz quantum cascade lasers operating at high temperatures

**DOI:** 10.1038/s41598-019-45957-8

**Published:** 2019-07-01

**Authors:** Li Wang, Tsung-Tse Lin, Ke Wang, Thomas Grange, Stefan Birner, Hideki Hirayama

**Affiliations:** 1 THz Quantum Device Team, RIKEN Center for Advanced Photonics, 519-1399 Aramaki-aza Aoba, Aoba-ku, Sendai, 980-0845 Japan; 2 School of Electronics Science and Engineering, Nanjing University, 163 Xianlin Street, Qixia District, Nanjing, 210046 China; 3 nextnano GmbH, Lichtenbergstr. 8, Garching bei München, 85748 Germany

**Keywords:** Optics and photonics, Engineering

## Abstract

Operating at high temperatures in the range of thermoelectric coolers is essential for terahertz quantum cascade lasers to real applications. The use of scattering-assisted injection scheme enables an increase in operating temperature. This concept, however, has not been implemented in a short-period structure consisting of two quantum wells. In this work, based on non-equilibrium Green’s function calculations, it emphasizes on the current leakage and parasitic absorption via high-energy states as fundamental limitations in this scheme with short-period. A new design concept employing asymmetric wells composition is proposed to suppress these limitations. A peak gain of 40 cm^−1^ at 230 K is predicted in the GaAs/AlGaAs semiconductor material system with an emission frequency of 3.5 THz.

## Introduction

Terahertz quantum cascade lasers (THz-QCLs)^1–5^ are emerging as promising sources of coherent THz wave. These semiconductor-based compact devices rely on optical intersubband (ISB) transitions in the periodic stack of quantum wells. The main hurdle for presently THz-QCLs is to enable its operating temperature within a range of thermoelectric coolers. A lot of efforts are devoted to determining the predominant mechanisms of temperature induced degradation^6–10^, and also to modify the active region designs^11–16^ for high-temperature tolerance. Thus far, the highest operating temperatures ever achieved in experiments (192K^17^, 199.5K^3^) are all based on resonant-phonon (RP) scheme, in which resonant tunneling (RT) mechanism is employed for injecting electrons into the upper laser level (ULL). However, RT injection has some inherent limitations, (i) the temperature-related dephasing can slow down RT^18,19^; (ii) a maximum population inversion is limited to only 50% of the total amount of electrons thanks to the reversibility of RT process.

Scattering-assisted injection (SAI) scheme is an attractive alternative for breakthrough. In contrast to RP scheme, SAI is free of restrictions on population inversion owing to the excess energy of injector state. The successful use of SAI concept was initially reported for mid-infrared QCLs^20,21^, and shortly after, it was applied to THz-QCLs based on triple-phonon scheme^22–24^. Theoretical studies indicate that, for THz-QCLs, SAI concept alone can increase the peak of optical gain (*g*_peak_) by a factor of three^25^. However, SAI THz-QCLs were only demonstrated by structures containing at least four wells in one period^22,24^. It is not yet verified in experiments with short-period structures (two or three quantum wells per period). Short length of period permits a larger fraction of electrons to contribute population inversions, thus much higher *g*_peak_ is expected. Indeed, the short-period structure is appealing for an increased operating temperature as evidenced in refs^3,17^ with RP scheme.

In this work, using non-equilibrium Green’s function calculations, the possibility of implementing SAI concept to THz-QCLs with short-period structure (two quantum wells) is investigated. It reveals that there are certain fundamental limitations in SAI two-well THz-QCLs, which originate from the high-energy states. Serious current leakage and also strong parasitic absorption are induced, thus resulting in a significant reduction of optical gain. In order to overcome these limitations, we propose a design adopting different alloy compositions in two quantum wells. In this way, the depth of wells can be tuned independently, and the detrimental high-energy confined states can be squeezed up. Based on a GaAs/AlGaAs material system, this proposed design allows us to get a *g*_peak_ as high as 40 cm^−1^ at 230 K, which is accessible to the compact thermoelectric coolers.

## Results and Discussion

The number of states involved in calculation is controlled by modifying the cut-off energy. First, only the lowest three basic states which are directly engaged in main transport, as represented by a term “[1, 2, 3]”, are allowed in calculation. The cut-off energy is then increased step by step in order to bring high-energy states one by one. For example, the term “[1, 2, 3] + [4]” means that state 4 is allowed in calculation, and so on. We first analyze the conventional SAI two-well THz-QCLs (design-1, barriers/wells: **2.7**/17.4/**1.25**/11.8 nm). It is a variant of an original structure (barriers/wells: **3**/17.5/**1.5**/11.5 nm) presented in ref.^26^. The laser frequency is deliberately shifted from 2.5 THz to 3.5 THz, at which frequency the high operating temperatures are mostly achieved in experiments^3,17^. With the purpose to suppress the parasitic injection channel, *i*.*e*., electrons flowing directly from injection level (IL) to lower laser level (LLL), the oscillator strength of laser states is set relatively small (~0.28). Design-2 is the proposed structure in which one period consists of two asymmetric wells: Al_0.04_Ga_0.96_As in the phonon well, instead of GaAs.

**Figure 1:**
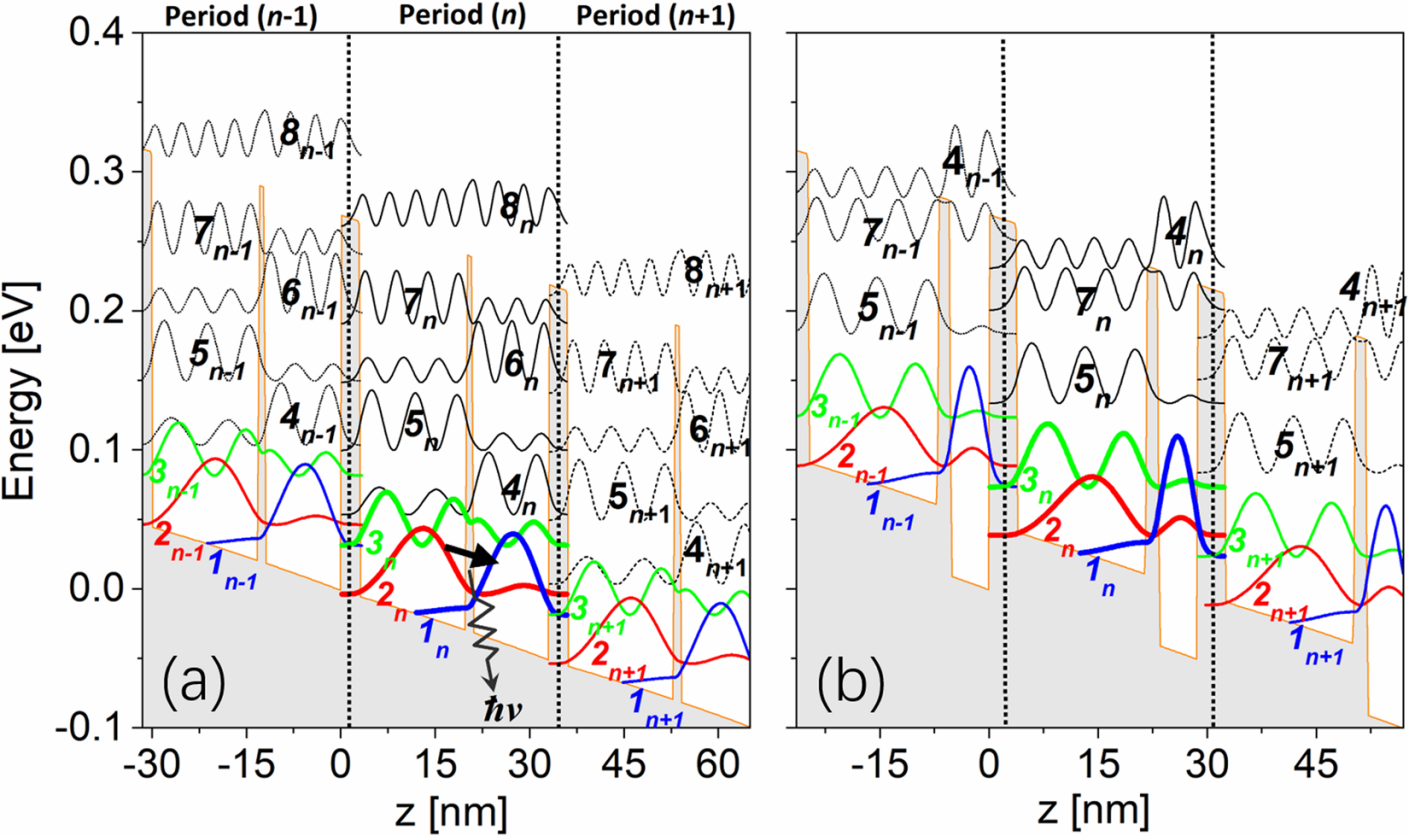
Conduction band diagram and tight-binding states of SAI two-well THz-QCLs with three neighboring periods *n* − 1, *n*, *n* + 1. (**a**) design-1 (Al_0.3_Ga_0.7_As/GaAs, barriers/wells: **2.7**/17.4/**1.25**/11.8 nm) and (**b**) design-2(Al_0.3_Ga_0.7_As/Al_0.04_Ga_0.96_As (GaAs), barriers/wells: **3.2**/17.9/**2**/5.3 nm). The underlined layer is doped by silicon at a sheet doping level of 3.9 × 10^10^ cm^−2^. Photon emission energy is 15 meV. Oscillator strength between laser states (ULL/LLL) is ~0.28.

Figure 1(a,b) show the conduction band diagrams and also squared envelope wave functions of design-1 and design-2 in the tight-binding basis. Three neighboring periods (*n* − 1, *n*, *n* + 1) are illustrated, and tunneling through the thicker barrier couples one period with its neighbor. Under the operating bias condition (52 mV/period), electrons fill ULL (state 2) from IL (state 3) by intrawell longitudinal-optical phonon (LO-phonon) scattering, then emit photons via ISB transitions from ULL to LLL (state 1). Electrons are finally extracted from LLL to the next IL in downstream period via resonant tunneling.

**Figure 2:**
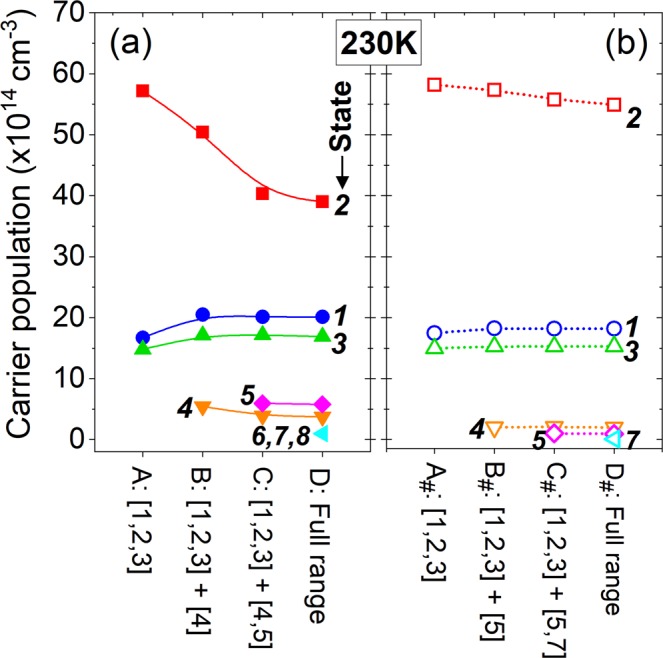
Population of each state in design-1 (**a**) and design-2 (**b**) at 230 K. It shows different cut-off energy under an operating bias condition (52 mV/period). The term “D: Full range” indicates the high-energy states 4, 5, 6, 7, 8 included in calculation.

**Figure 3:**
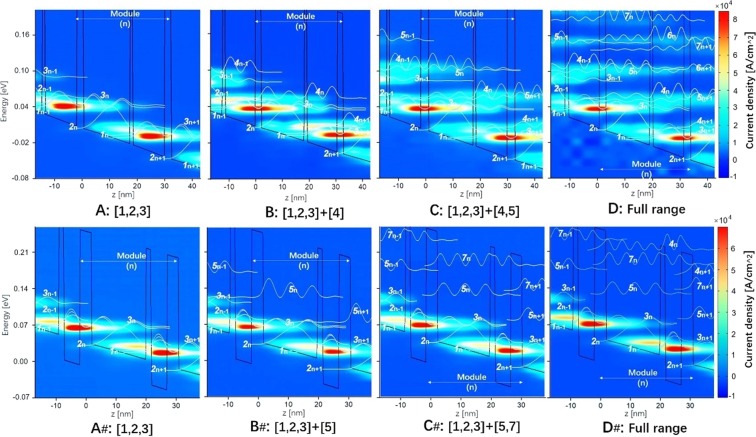
Position-energy resolved current density mappings at an operating bias of 52 mV/period for both design-1 (up-row, A, B, C, D) and design-2 (down-row, A_#_, B_#_, C_#_, D_#_), with various high-energy states involved one by one.

**Figure 4:**
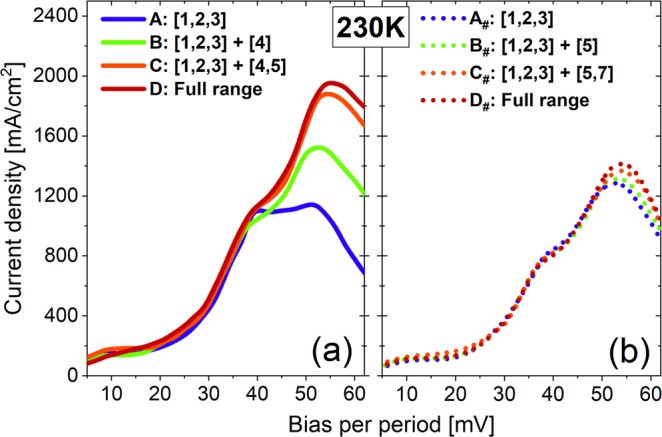
Plots of current density versus voltage for both design-1 (**a**) and design-2 (**b**) at 230 K, with different numbers of high-energy states included in calculations.

Figure 2(a) shows the number of electrons (population) at each state in design-1 at 230 K. A, B, C, D letters in lateral axis indicate the calculations including just three basic states, or basic states plus high-energy states one by one. A significant decrease in the population of ULL (*n*_2_), by a factor of 1/3, can be found, if all high-energy states included (D: Full range), while the population of LLL (*n*_1_) increases slightly. It therefore leads to a considerable reduction of population inversion Δ*n*_21_ (Δ*n*_21_ = *n*_2_ − *n*_1_). It is obvious from the energy-position resolved mappings of current density as shown in Fig. 3 (up-row: A, B, C, D), the decrease in *n*_2_ is attributed to severe current leakage via high-energy states 4 and 5. Leakage channels can be formed as there are strong sequential tunnels between ULL in period *n*-1 (state 2_*n*-1_), state 4_*n*_ in the first downstream period *n*, and state 5_*n*+1_ in the second downstream period *n* + 1. The reason is that, these three states are quite close energetically and also spatially. In design-1, originating from the laser barrier be thin, state 4 is strongly delocalized. The sequential tunnels via states 2_*n*-1_, 4_*n*_ and 5_*n*+1_, as a result, will be efficient as it only tunnels through one extraction barrier (at the boundary of neighboring periods). The corresponding tunnel coupling strength for pairs of states (2_*n*-1_ ↔ 4_*n*_, 4_*n*_ ↔ 5_*n*+1_, and 2_*n*-1_ ↔ 5_*n*+1_) are 2.6 meV, 5.1 meV, and 1.2 meV, respectively. It agrees well with the fact that current leakages extending over at least three neighboring periods as shown in Fig. 3 (up-row). The current-voltage plots in Fig. 4(a) show more evidences that, at operating bias, current peak increases appreciably if both states 4 and 5 allowed in calculation. Inclusion of more high-energy states (states 6, 7, 8) increases the current density further, but very slightly. These results are consistent with the analysis above. It therefore can be concluded here, states 4 and 5 that belong to different neighboring periods are crucial to the serious current leakages. In addition, the increase in population of LLL (*n*_1_) is ascribed to another parasitic channel inside one single period (state 3 → 4 → 1) via phonon resonance, in which state 4 also plays a detrimental role.

**Figure 5:**
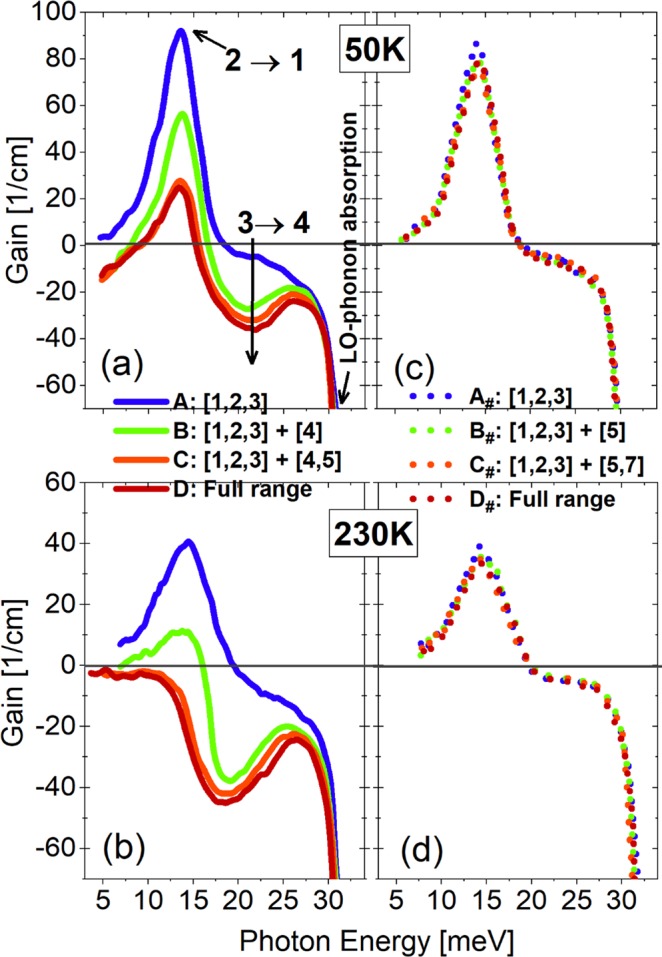
Calculated optical gain spectra under an operating bias of 52 mV/period for design-1 (**a**,**b**) and design-2 (**c**,**d**) at both low and high temperatures (50 K; 230 K), respectively. High-energy state is involved one by one.

The predominant harmful impacts of both states 4 and 5 on lasing can be quantitatively estimated from the changes in optical gain, which is one of the key parameters for THz-QCLs. Figure 5(a,b) show the optical gain spectra of design-1 at both low and high temperatures (50 K, 230 K). When state 4 is included (curve B), the *g*_peak_ at 50 K is reduced from 93 cm^−1^ to 57 cm^−1^, and at 230 K, it decreases from 42 cm^−1^ to 17 cm^−1^. If both states 4 and 5 are included (curve C), the *g*_peak_ at 50 K reduces further to 27 cm^−1^, and it becomes astonishingly negative at 230 K. As mentioned above, the decrease in population inversion Δ*n*_21_ due to leakage could be responsible for such reduction in optical gain. However, in case of 230 K, the change of population inversion Δ*n*_21_ cannot fully explain the turn of *g*_peak_ from positive to negative. In fact, when both states 4 and 5 are included, the *g*_peak_ at 230 K becomes -19 cm^−1^, but Δ*n*_21_ only decreases by half.

**Figure 6:**
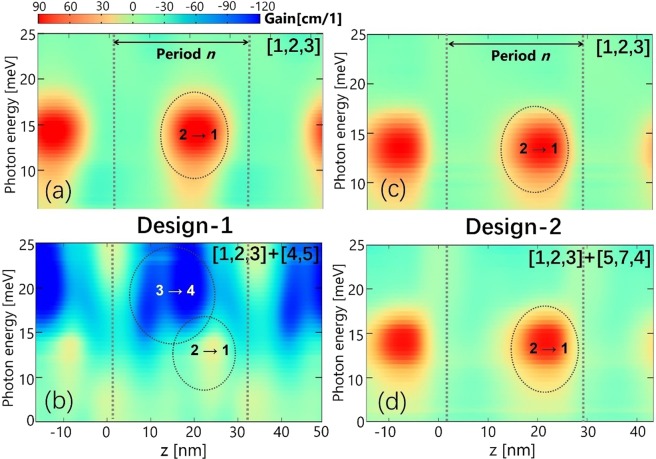
Position-energy resolved gain mappings for design-1 (**a**,**b**) and design-2 (**c**,**d**) at 230 K under an operating bias of 52 mV/period.

In this work, we find that the further reduction of *g*_peak_ to negative is due to a parasitic absorption inside one single period. Figure 5(b) indicates that a strong parasitic absorption between states 3_*n*_ and 4_*n*_, which is centered at 20 meV, emerges, and overlaps closely with the radiative transition (states 2 → 1). State 5_*n*-1_ in downstream period can efficiently depopulate state 4_*n*_ and enhances this absorption. The dipole matrix elements of states 3_*n*_ and 4_*n*_ (both excited states) are quite large (6.5 nm), in contrast to that of 3.4 nm between 2_*n*_ and 1_*n*_ (ULL/LLL). In addition, at high temperature of 230 K, thermal backfilling from state 2_*n*_ to 3_*n*_ will become noticeable, which further make this absorption stronger. Position-energy resolved gain mappings, as shown in Fig. 6(a), reveal very intuitive evidences of this absorption and also the overlaps. Just considering three basic states, positive gain (states 2 → 1) is clearly demonstrated. When both states 4 and 5 are included, absorption (states 3 → 4) can be observed from Fig. 6(b). This absorption area is quite broad and closely overlaps with the positive gain area, thus resulting in the *g*_peak_ turning negative.

To overcome these limitations in design-1, we propose to use different alloy composition in two quantum wells (design-2 with asymmetric wells). In this way, as shown in Fig. 1(b), the lower laser well can be narrowed independently, and state 4 in this well will be squeezed up very high in energy, even above the confinement potential into the continuum. States in the phonon well also have to be tuned up accordingly. This is done by replacing GaAs well with Al_0.04_Ga_0.96_As well, in which the conduction band edge is about 36 meV higher than that of GaAs, if assuming the conduction band offset in GaAs/Al_0.3_Ga_0.7_As be 270 meV.

Current leakages are almost inhibited as indicated in the mappings of energy-position resolved current density shown in Fig. 3 (down-row: A_#_, B_#_, C_#_, D_#_) and also the current-voltage plots in Fig. 4(b). It confirms the importance of suppressing multi-period leakages via state 4. As discussed above, state 5 is also very important for the leakages, but it is hardly tuned since it is the second excited state in the phonon well. But the interaction of ULL and state 5 in second downstream period (2_*n*-1_ ↔ 5_*n*+1_) is very weak without the “bridge” state 4_*n*_. Actually, these two states are spatially far away, by two periods, to be an active leakage channel. Consequently, the channels of sequential multi-period leakages are effectively blocked just by squeezing up state 4. The population inversion Δ*n*_21_ changes very little as shown in Fig. 2(b). Optical gain spectra of design-2 are almost identical regarding just three basic states and plus high-energy states as shown in Fig. 5(c,d). Concerning the parasitic absorption discussed in design-1, the lifting-up of state 4 totally suppresses it, since the energy separation between states 3 and 4 becomes very large (184 meV). This is clearly confirmed by the optical gain spectra in Fig. 5(c,d) and also the mappings of position-energy resolved gain in Fig. 6(c,d).

The limitations in design-1 are difficult to be suppressed by only optimizing the thicknesses of barriers and wells, because of a lack of tuning freedom. The possible tuning space in phonon well is strictly limited due to a need of LO-phonon energy separation between states 3 and 2. The lower laser well is then set by a designed photon emission energy, so the first excited state in this well (state 4), which is shown to be highly detrimental, is hardly tuned in energy. Also, each period of SAI two-well THz-QCLs is with a short length, and it is easy to bring tunneling via state 4 to the neighboring periods.

The asymmetric wells composition concept is not limited to the design discussed in this work. By offering more design freedom, it also could be applied to three- or four-well THz-QCLs, which may also suffer some negative impacts from high-energy states, especially at high operating temperatures. This concept could also be applied in other material systems, *e*.*g*., non-polar n-type Ge/SiGe in which current leakages and parasitic absorption are expected to be stronger^27^.

## Conclusion

In summary, for short-period (two-well) SAI THz-QCLs, limitations imposing by high-energy states are systematically studied. The first excited state in lower laser well is identified to be mainly responsible for a dramatic reduction in optical gain. This high-energy state induces severe detrimental leakages that tunnel to several downstream periods, as well as strong parasitic absorption. Two-well design with asymmetric wells composition is demonstrated giving more design freedom that the high-energy states can be tuned independently. In this way, the detrimental effects of high-energy state are significantly suppressed. A high peak gain of 40 cm^−1^ is predicted in the GaAs/AlGaAs material system at a high operating temperature of 230 K.

## Method

The transport and optical gain properties are quantitatively calculated by using the non-equilibrium Green’s function (NEGF) formalisms^25,28–32^ implementing in the nextnano.QCL simulation package. The NEGF method is capable to reveal the full quantum transport within cascading quantum wells structure. This method has demonstrated its reliability of modelling the dephasing that arises from charged impurity scattering^33^. The basis-invariant description in this model enables accurate predictions of the sequential leakage channels via high-energy states, which is one main issue in this work. In contrast, it is more challenging for the methods of rate equations, Monte Carlo, or density-matrix to address such leakage current. Electron-electron interactions are treated as an elastic approximation^34^. Non-elastic (electron-phonon interactions) and elastic (charged impurity, interface roughness) scatterings are also considered in this package, and for alloy disorders, since Al_0.04_Ga_0.96_As is used for phonon well, the alloy scattering involved in transport is as a function dependent on its position. More details of scatterings are described in refs^29,31,32^. In addition, in contrast to the NEGF studies in previous reports^35,36^, in-plane momentum dependence of the scattering matrix element is employed in our package.
